# Molecular identification of native *Wolbachia pipientis* in *Anopheles minimus* in a low-malaria transmission area of Umphang Valley along the Thailand-Myanmar border

**DOI:** 10.1186/s13071-020-04459-7

**Published:** 2020-11-16

**Authors:** Nongnat Tongkrajang, Pichet Ruenchit, Chatchai Tananchai, Theeraphap Chareonviriyaphap, Kasem Kulkeaw

**Affiliations:** 1 Department of Parasitology, Faculty of Medicine Siriraj Hospital, Mahidol University, The 7th floor, Adulyadejvikrom Building, 2 Wang Lang Road, Bangkok-Noi, Bangkok, 10700 Thailand; 2 Department of Entomology, Faculty of Agriculture, Kasetsart University, 2nd floor, Jarad Sunthornsingh Building, 50 Ngam Wong Wan Road, Chatuchak, Bangkok, 10900 Thailand

**Keywords:** *Wolbachia*, *Anopheles*, Malaria, 16S rRNA, PCR, Phylogenetics

## Abstract

**Background:**

*Wolbachia*, obligate intracellular bacteria, infect the majority of arthropods, including many mosquito species of medical importance. Some *Wolbachia* strains interfere with the development of *Plasmodium* parasites in female *Anopheles*, a major vector of malaria. The use of *Wolbachia* as a means to block malaria transmission is an emerging vector control strategy in highly endemic areas. Hence, identification of native *Wolbachia* strains in areas where malaria transmission is low may uncover a particular *Wolbachia* strain capable of *Plasmodium* interference. This study aims to identify native *Wolbachia* strains in female *Anopheles* spp. that are predominant in a low-malaria transmission area in mainland Southeast Asia.

**Methods:**

Following a 2-year survey of malaria vectors in Umphang Valley of Tak Province, Thailand, DNA extracts of female *An. minimus*, *An. peditaeniatus*, and *An. maculatus* were subjected to amplification of the conserved region of the 16S rRNA-encoding gene. The DNA sequences of the amplicons were phylogenetically compared with those of known *Wolbachia* strains.

**Results:**

Among three *Anopheles* spp., amplification was detected in only the DNA samples from *An. minimus*. The DNA sequencing of amplicons revealed 100% similarity to *Wolbachia pipientis*, confirming the specificity of amplification. The *Wolbachia*-positive *An. minimus* samples were devoid of *Plasmodium* 18S rRNA amplification. The phylogenetic trees indicate a close relationship with *Wolbachia* strains in subgroup B.

**Conclusion:**

To the best of our knowledge, the data presented herein provide the first molecular evidence of a *Wolbachia* strain in *An. minimus*, hereinafter named *w*Anmi, in a low-malaria transmission area in the Umphang Valley of western Thailand. Further biological characterization is required to examine its potential for malaria transmission control in the field.

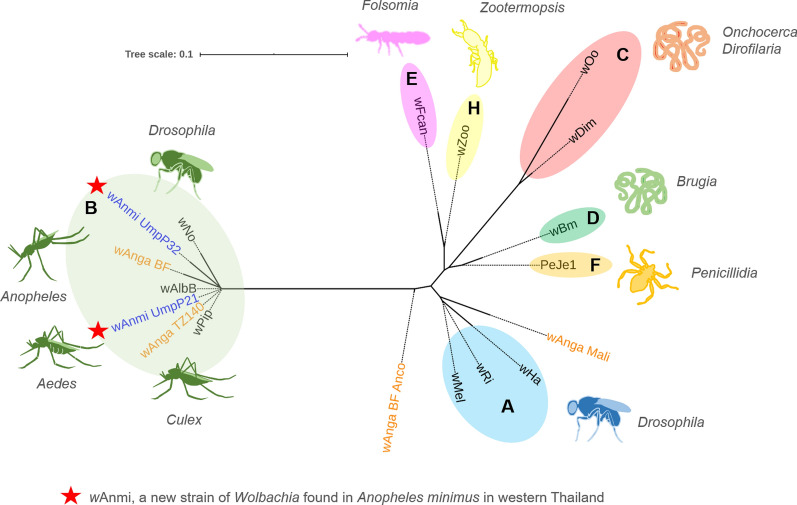

## Background

Malaria is a vector-borne parasitic disease caused by *Plasmodium* species. Ongoing malaria control programs significantly decreased morbidity and mortality in Africa and Asia between 2010 and 2018 [1]; however, many parts of the border regions, especially in Southeast Asia, are still malaria-endemic areas [1, 2]. Given that *Anopheles* mosquitoes are malaria vectors, one of the effective strategies to control malaria transmission relies on the use of insecticides, including indoor residual spraying and the use of insecticide-treated mosquito nets. Accordingly, resistance to insecticides has emerged as a biological threat to malaria control and elimination efforts in endemic areas, including many regions located in forest-mountain landscapes along the Thailand-Myanmar [3] and Thailand-Laos PDR borders [4]. Although widespread insecticide resistance has increased globally in many malaria-endemic regions, there was no evidence of an association between insecticide resistance and malaria burden [5]. However, a meta-analysis-based model of malaria transmission predicted that insecticide resistance potentially increased malaria incidence in part because of the decrement of mosquito mortality [6], challenging global malaria eradication. Thus, an effective alternative to insecticides is needed.

*Wolbachia* is an intracellular bacterium that naturally infects the majority of insect species [7]. *Wolbachia* bacteria reside in the cytoplasmic vacuoles of various types of insect somatic and germ cells, allowing maternal transmission to their progeny. *Wolbachia* is capable of manipulating host reproduction through cytoplasmic incompatibility [8, 9], in which *Wolbachia*-infected eggs form viable offspring and noninfected eggs do not. Moreover, the infected progeny tend to sexually develop into females, while unfertilized eggs develop into females [10, 11], leading to widespread *Wolbachia* infection in an insect population [12, 13], including mosquito species of medical importance. Since *Wolbachia* is reportedly capable of suppressing pathogen development and blocking disease transmission, the use of *Wolbachia* has been proposed as a mean of controlling transmission of pathogenic viruses causing dengue virus [14, 15], West Nile virus [16], yellow fever virus, and Chikungunya virus [17].

Given failures in the detection of *Wolbachia* in *Anopheles* mosquitoes, it was initially hypothesized that *Anopheles* mosquitoes are refractory to *Wolbachia* infection [18-21]. However, a study demonstrated that the *Aedes albopictus*-specific *Wolbachia* strain AlbB (*w*AlbB) could infect laboratory-reared *An. stephensi* and suppress the development of *P. falciparum* within female *Anopheles* mosquitoes [22]. In concordance with the laboratory study, *Wolbachia* infections were observed in natural populations of *An. gambiae* and *An. coluzzii*, two major vectors in malaria-endemic regions of Burkina Faso [23, 24]. Interestingly, researchers phylogenetically identified *Anopheles*-infecting *Wolbachia* as a new arthropod-specific subgroup named *w*Anga [23]. Previous reports have shown evidence of natural *Wolbachia* endosymbiosis in other *Anopheles* species as well as its effects on *Plasmodium* development. In the high-malaria transmission area of Burkina Faso, a field study showed that natural infection with the *Wolbachia* strain *w*Anga in blood-fed *An. coluzzii* females was negatively correlated with *Plasmodium* development [25]. Based on a mathematical model, natural *Wolbachia* infection potentially blocks malaria transmission from vector to human [25]. Furthermore, infection by the *Wolbachia* strain wAnga-Mali in *An. gambiae* was associated with a reduced prevalence and intensity of sporozoite infection in field-collected females in Mali [26]. Altogether, studies strongly suggest that *Anopheles* mosquitoes are permissible to *Wolbachia* endosymbiosis and that some strains of *Wolbachia* are capable of interfering with the development of *Plasmodium* parasites in female *Anopheles*. Thus, the release of laboratory-reared, *Wolbachia*-infected *Anopheles* mosquitoes to replace the wild *Anopheles* population is a potential strategy to block malaria transmission. Hence, identification of native *Wolbachia* strains in areas where malaria transmission is low may uncover a particular *Wolbachia* strain capable of interfering with *Plasmodium* development in *Anopheles*.

In Thailand, only one survey of *Wolbachia* in mosquitoes was conducted to amplify the *filamenting temperature-sensitive mutant Z* (*ftsz*) and *Wolbachia surface protein* (*wsp*) genes. All 23 mosquito species in the genera *Aedes*, *Culex*, and *Mansonia* were positive for the *ftsz* and *wsp* genes, whereas none of the 19 *Anopheles* species were positive [18]. Failure to detect *Wolbachia*-specific genes in *Anopheles* spp. was consistent with the results of studies in European, African, and American specimens [19, 20]. Nevertheless, detection of the *Wolbachia* 16S rRNA region was accomplished. The W-Spec primers were designed to specifically amplify a 438-bp sequence at the 3ʹ region of the 16S rRNA gene in *Wolbachia* [27]. The W-Spec primers allowed the detection of *Wolbachia* in temperate North American arthropods, including the family *Culicidae* but excluding other mosquito families. Subsequently, Baldini et al. reported the first evidence of *Wolbachia* in the reproductive organs of male and female *An. gambiae*, a major malaria vector in sub-Saharan Africa. In the same DNA samples, the W-Spec primer-based PCR was able to amplify the 16S rRNA fragment, whereas *Wolbachia*-specific surface protein and fructose-biphosphate aldolase-based PCR failed [23], implying good sensitivity of the W-Spec primers. Moreover, Shaw et al. further improved the sensitivity of W-Spec primer-based PCR by using nested primers (16SNF and 16SNR). The use of nested PCR allowed the detection of *Wolbachia* in *An. coluzzii* [25], *An. gambiae* in Mali [26], and *An. arabiensis* in Tanzania [28]. Additional studies were able to amplify the *Wolbachia* 16S rRNA fragment in DNA samples extracted from head-thorax or thorax-abdomen, implying the possibility of *Wolbachia* infection in nonreproductive organs [22, 29]. Collectively, *Wolbachia* infection in somatic and germ cells can be detected using nested PCR, which amplifies the conserved region of the *Wolbachia* 16S rRNA gene. Considering the availability of DNA extracts from major *Anopheles* species obtained during a 2-year survey of malaria vectors [30] and *An. minimus* is the important malaria vector carrying *P. vivax* sporozoites [31], this study aims to identify native *Wolbachia* strains in female *Anopheles* spp. that are predominant in a low-transmission area in Umphang Valley, located near the Thailand-Myanmar border of mainland Southeast Asia.

## Methods

### Biosafety for using biological samples of mosquitoes

The protocol for the use of DNA samples extracted from *Anopheles* mosquitoes was approved by the Siriraj Safety Risk Management Taskforce, Faculty of Medicine Siriraj Hospital, Mahidol University (SI2020-010). In accordance with the guidelines for ethics in animal use, this study submitted the DNA extraction protocol and sampling details to the Siriraj Animal Care and Use Committee, Faculty of Medicine Siriraj Hospital, Mahidol University (COA 012/2563), and received permission.

### Collection and identification of Anopheles species

**Fig. 1:**
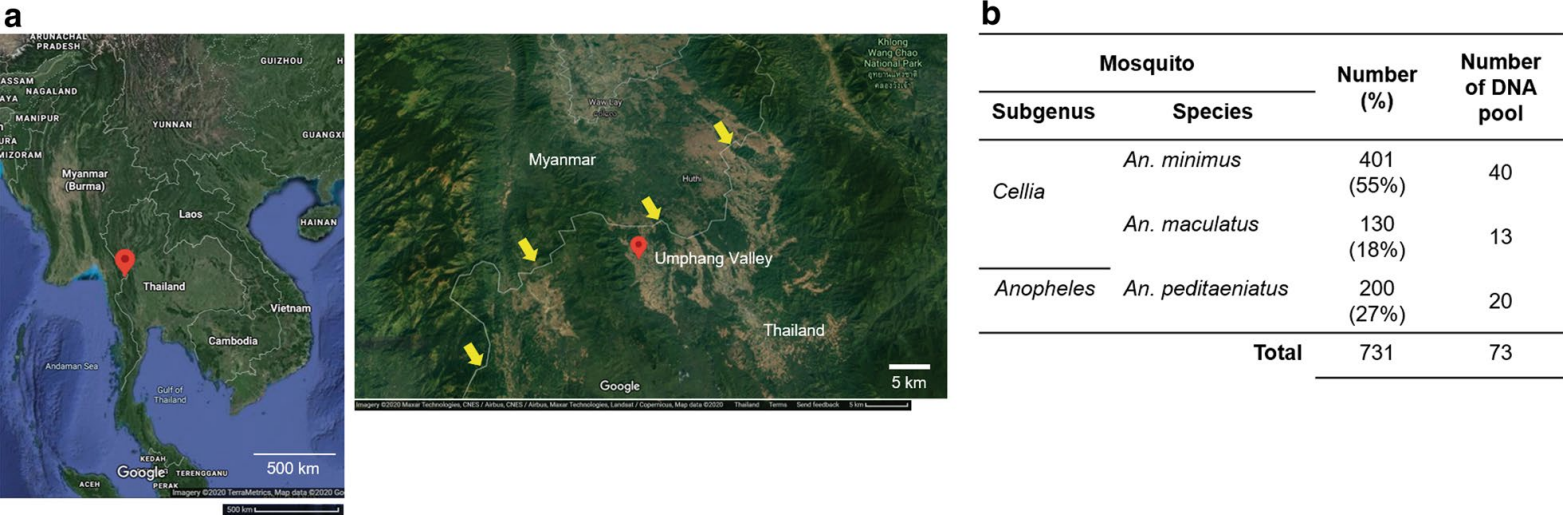
Collection site of *Anopheles* spp. **a** Google map of mainland Southeast Asia (left) and the location in which the *Anopheles* spp. were collected (right side). The collection site was located at 16° 04ʹ 36.3ʺ N 98° 45ʹ 8.0ʺ E (red pins) in Umphang Valley of Tak Province in western Thailand. The yellow arrow indicates the Thailand-Myanmar border. Scale bars are 500 and 5 km. **b** *Anopheles* spp. in the subgenera *Cellia* and *Anopheles* and the total number of DNA samples included in this study

Since this study is an extension of a previously published report, we did not collect and identify *Anopheles* species. For detailed collection methods, we highly recommend reading the original article [30]. The collection site was located in Ban Nong Luang village (16° 04ʹ 36.3ʺ N 98° 45ʹ 8.0ʺ E), Umphang District of Tak Province, located in western Thailand (Fig. 1a). The village is located approximately 4 km from the border of Kayin state, Myanmar. Mosquitoes were captured for five consecutive nights every 2 months from February 2015 to December 2016. The standard mosquito landing collection procedure followed that in a previous report [32]. Briefly, mosquito capture methods included indoor human landing, outdoor human landing, and cattle-baited outdoor collections. For human landing, mosquitoes were collected in a 6-h period: 18:00-00:00 h and 00:00-06:00 h. In indoor human landing, a volunteer was sitting inside the house. When mosquitoes landed on the lower part of the legs, they were collected using aspiration. The collection was performed continuously for 45 min, followed by a 15-min resting period. To collect mosquitoes outdoors, another volunteer stayed outside the house located 30 m away from the same house. For the cattle-baited outdoor collections, an adult cow was covered with a two-layered cotton bed net. To prevent mosquito biting, the inner layer enclosed the ground, while the outer layer was above the ground, allowing entry of mosquitoes into the net. Interlayer-residing mosquitoes were collected at the end of each 45-min collection period. Mosquitoes were identified following a standard dichotomous key [33], and *Mansonia* mosquito were morphologically identified according to the key characteristics [34]. Individual mosquitoes were placed in a 1.5-ml tube, frozen in liquid nitrogen, and stored at − 80 °C. For molecular identification of *Anopheles* spp. and *Wolbachia*, DNA from individual mosquitoes was extracted from the head and thorax and subjected to the multiplex allele-specific polymerase chain reaction assays and nested PCR, respectively. There were four assays of multiplex allele-specific PCR with regard to the Dirus Complex (*An. dirus*, *An. cracens*, *An. scanloni*, *An. baimaii*, and *An. nemophilous*) [35], the Minimus Complex and related species (*An. minimus*, *An. harrisoni*, *An. aconitus*, *An. varuna* and *An. pampanai*) [36], the Maculatus Group (*An. maculatus*, *An. sawadwongporni*, *An. pseudowillmori*, *An. dravidicus* and *An. rampae* (former Form K)) [37], and the Hyrcanus Group (*An. argyropus*, *An. crawfordi*, *An. nigerrimus*, *An. nitidus*, *An. paraliae*, *An. peditaeniatus*, *An. pursati*, and *An. sinensis*) [38]. A total of 731 DNA samples were obtained from *An. minimus* (*n* = 401, 55%), *An. peditaeniatus* (*n* = 200, 27%), and *An. maculatus* (*n* = 130, 18%) (Fig. 1b). To pool the DNA from each mosquito species for analysis, 2 μL of 10–11 DNA samples was mixed in the same tube. There were 40, 20, and 13 DNA sample pools from *An. minimus*, *An. peditaeniatus*, and *An. maculatus*, respectively (Fig. 1b).

### Amplification of the Wolbachia-specific 16S rRNA coding region

**Fig. 2:**
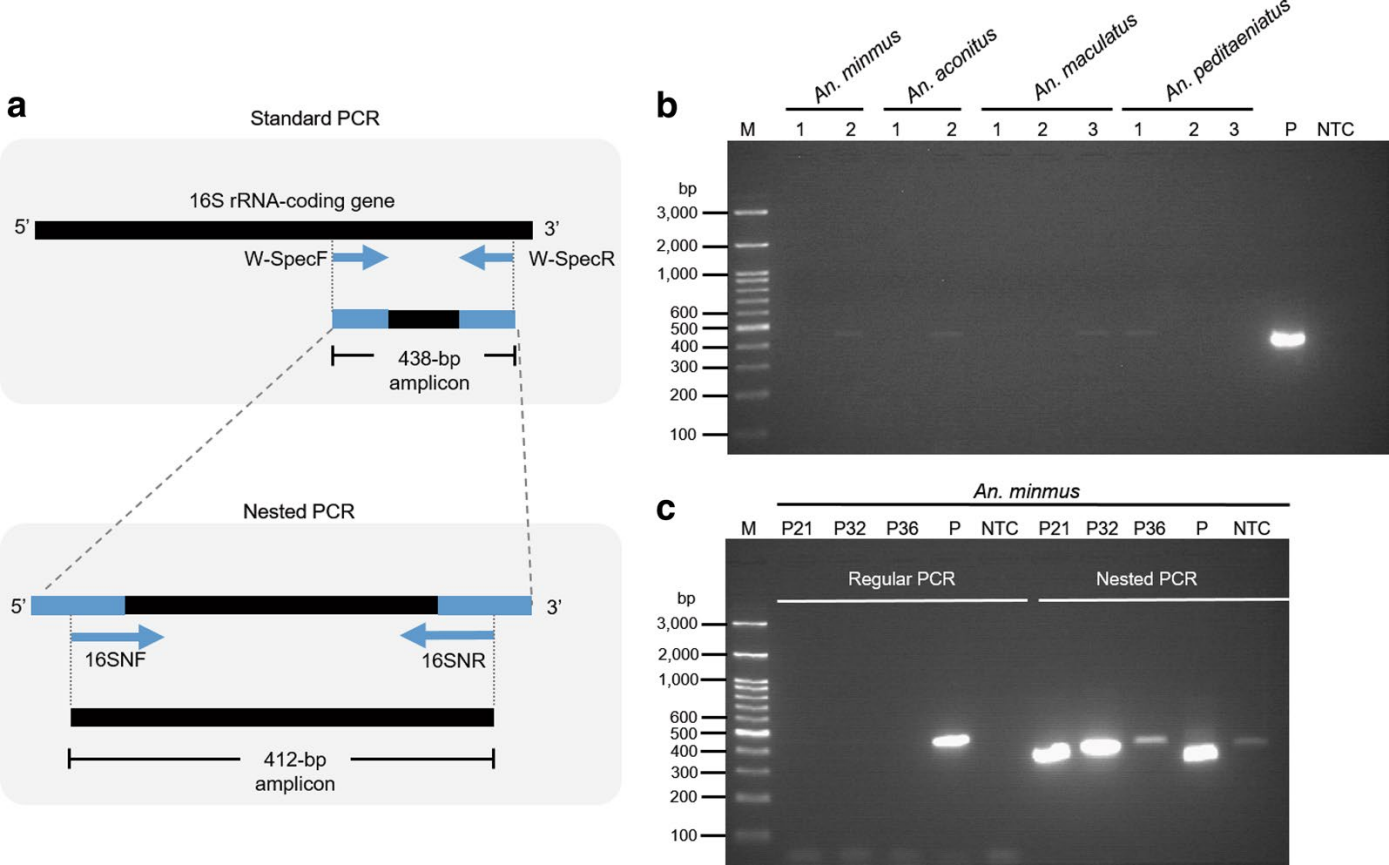
Amplification of the *Wolbachia* 16S rRNA-encoding gene. **a** Schematic diagram showing two-step PCR, including standard and nested PCR. In the standard PCR, W-SpecF and W-SpecR primers (blue colored arrows in upper panel) attached to the 3ʹ region of the Wolbachia 16S rRNA-encoding gene, amplifying a 438-bp fragment. In the nested PCR, the 438-bp amplicons generated from the regular PCR were used as templates. The 16SNF and 16SNR primers attached the internal sequence of the 438-bp fragment, generating a 412-bp PCR product. **b** A representative image of the 438-bp amplicons obtained from the standard PCR. Two and three representative pools of DNA extracts of *Anopheles* are shown. **c** A representative image of amplicons derived from the standard PCR (438 bp) and nested PCR (412 bp). For the standard PCR, templates were obtained from the DNA extracts from *An. minimus* pool numbers 21 (P21), 32 (P32), and 36 (P36). DNA from *Mansonia* spp. was used as the positive control (P), while absent template DNA was used as the negative control (NTC). In the nested PCR, all the samples from the standard PCR were used as templates. The PCR products were analyzed with electrophoresis in a 2% agarose gel. Lane M: the DNA ladder electrophoresed simultaneously with the PCR product to determine amplicon size

To amplify a conserved region of the *Wolbachia* 16S rDNA-encoding gene, W-SpecF and W-SpecR primers were used in the initial standard PCR, and 16SNF and 16SNR primers were used in the nested PCR, following a previous report [27] (Fig. 2a). Primer sequences are shown in Table 1. Samples were prepared with a total volume of 10 µL, which was composed of 0.5 μM of each primer and 1 μL of DNA template and AccuStart™ II Gel Track PCR SuperMix (Quantabio, Beverly, MA, USA). Amplification was performed with DNA template denaturation at 95 °C for 3 min; followed by 35 cycles of DNA denaturation at 95 °C for 15 s, primer annealing at 50 °C for 25 s, and DNA extension at 72 °C for 30 s; and final extension at 72 °C for 5 min. To visualize the DNA bands, the PCR products were mixed with ViSafe Red (Vivantis Technologies Sdn. Bhd., Selangor Darul Ehsan, Malaysia) and subsequently electrophoresed in 2% agarose gel in 1× TAE buffer at a voltage of 100 V for 45–50 min. The ViSafe Red-intercalated, double-stranded DNA sequences were then exposed to UV light (Molecular Imager^®^ Gel Doc™ XR System, Bio-Rad Laboratories, Inc., Hercules, CA) for visualization. The length of the amplicon yielded from the initial PCR was approximately 438 bp in length. Subsequently, 1 µL of the initial PCR product was used as template for the nested PCR, in which 16SNF and 16SNR primers to bind to the internal sequence of W-SpecF and W-SpecR were added (Fig. 2a, lower panel). The thermal cycles included initial denaturation at 95 °C for 3 min; followed by 35 cycles of denaturation at 95 °C for 15 s, primer annealing at 60 °C for 25 s and extension at 72 °C for 30 s; with a final extension at 72 °C for 5 min. The length of the amplicon yielded from the nested PCR was approximately 412 bp in length. To confirm specificity, the 412-bp amplicons were purified from the agarose gel and sequenced by an ABI 3730XL DNA Analyzer (Bionics, Seoul, South Korea). The 16SNF and 16SNR primers were used as DNA sequencing primers. Since *Mansonia uniformis* and *M. indiana* are naturally infected with *Wolbachia* [18, 21] in Thailand, DNA extracts of *Mansonia* mosquitoes were used as the positive control. PCR without DNA template was used as the negative control.

**Table 1:** Primers used in this study

Type	Targets	Primer names	Primer sequence (5ʹ–3ʹ)
Nested PCR	*Wolbachia* 16S rRNA	W-SpecF	CATACCTATTCGAAGGGATAG
		W-SpecR	AGCTTCGAGTGAAACCAATTC
		16SNF	GAAGGGATAGGGTCGGTTCG
		16SNR	CAATTCCCATGGCGTGACG
qPCR	*Plasmodium* 18S rRNA	PlasF	CTTAGTTACGATTAATAGGAGTAGC
		PlasR	GAAAATCTAAGAATTTCACCTCTGA
	*Wolbachia* 16S rRNA	W-SpecF	CATACCTATTCGAAGGGATAG
		W16S	TTGCGGGACTTAACCCAACA
	*An. minimus* ITS2A	ITS2A	TGT GAA CTG CAG GAC ACA T
		MIA	CCC GTG CGA CTT GAC GA

### Quantitative amplification of *Wolbachia* 16S rRNA and *Plasmodium* 18S rRNA region

Level of *Wolbachia* 16S rRNA- and *Plasmodium* 18S rRNA-coding DNA sequence was examined in the same DNA sample as the nested PCR using quantitative real-time PCR (qPCR). The use of W-Specf and W16S primers in qPCR had > 100% efficiency in amplification and detected the *Wolbachia* 16S rRNA-coding region at concentrations lower than combination of W-Specf and W-Specr did [26, 39]. Thus, this study deployed the W-Specf and W16S primers. Detection of *Plasmodium* 18S rRNA and An. minimus ITS2A region was performed using the primer set published by Shaw et al. [25] and Eamet al. [40]. Primer sets are shown in Table 1. Luna^®^ Universal qPCR Master Mix (New England BioLabs) was used, and the primer concentration was 250 nM for each primer. After an initial denaturation at 95 °C for 1 min, thermal cycles were as follows: denaturation at 95 °C for 15 s and annealing and extension at 60 °C for 30 s (CFX96™ Real-Time System and C1000™ Thermal Cycler, Bio-Rad). The amplicons having melting temperatures similar to the positive control were interpreted as “detectable,” whereas amplicons that have different melting temperature or cycle threshold (Ct) values > 40 were regarded as “undetectable” [26]. To compare the amount of gDNA template of *Wolbachia* and *Plasmodium* spp., the level of *Wolbachia 16S* rRNA- and *Plasmodium* 18S rRNA-coding DNA sequence was normalized with the level of the *An.* *minimus* ITS2A region. Levels of *Wolbachia* and *Plasmodium* spp. in a given sample were compared using the 2^−∆∆CT^ method based on assumption of 100% qPCR efficiency and were shown as the relative level of *Wolbachia* [41]. Gene expression analyses were carried out in triplicate for each sample. The gDNA of *Mansonia* spp. or *Plasmodium falciparum* strain K1 [42] were used as positive control, respectively. Nuclease-free water was set as the no template control. Melt cure analysis was performed at the end of amplification.

### Bioinformatics

The obtained sequences of the *Wolbachia* 16S rRNA fragment were edited and assembled using BioEdit Sequence Alignment Editor (version 7.2.5). DNA sequences were deposited in GenBank (accession nos. MT449018 and MT449019). To identify similar sequences, the GenBank database was searched with BLASTN [43]. For sequence alignment, the following sequences of the 16S rRNA-encoding gene of *Wolbachia* subgroup B were obtained: *Wolbachia* strain *w*No from *Drosophila simulans* (CP003883.1), strain *w*AlbB from *Ae. albopictus* (KX155506.1), and *w*Pip from *Culex quinquefasciatus* (AM999887.1). To analyze nucleotide substitution, the multiple sequence alignment was performed using MSAViewer.

### Phylogenetic analysis

The conserved region of the *Wolbachia* 16S rRNA-encoding gene was phylogenetically analyzed using Molecular Evolutionary Genetics Analysis (MEGA) software version 10.0 [44] and NGPhylogeny.fr [45]. *Wolbachia* 16S rRNA sequences of other strains belonging to subgroups A, B, C, D, E, F, H, and *Anopheles*-specific subgroups were retrieved from GenBank for analysis (Table 2). Rooted and unrooted phylogenetic trees were analyzed based on the neighbor-joining method. *Rickettsia montanensis* was used as a non-*Wolbachia* outgroup for rooted tree analysis.

**Table 2:** Sources of *Wolbachia* 16S rRNA sequences used in this study

Subgroup	Strain	Natural *Wolbachia* host	Common name	NCBI Accession numbers for 16S rRNA sequences
A	wMel	*Drosophila melanogaster*	Fruit fly	AE017196
	wRi	*Drosophila simulans*		CP001391
	wHa	*Drosophila simulans*		CP003884
B	wPip	*Culex quinquefasciatus*	Southern house mosquito	AM999887.1
	wAlbB	*Aedes albopictus*	Asian tiger mosquito	KX155506.1
	wNo	*Drosophila simulans*	Fruit fly	CP003883.1
C	wOo	*Onchocerca ochengi*	Filarial nematode of cattle	AJ010276.1
	wDim	*Dirofilaria immitis*	Heartworm of dogs	AF487892.1
D	wBm	*Brugia malayi*	Filarial nematode of humans	AJ010275
E	wFcan	*Folsomia candida*	Springtail	KT799585.1
F	PeJe1	*Penicillidia jenynsii*	Wingless bat fly	AB632590
H	wZoo	*Zootermopsis nevadensis*	Termite	AY764280
Anopheles-specific	wAnga_BF_Anco	*Anopheles coluzzii*	Common malaria mosquito	KP089991
	wAnga_BF	*Anopheles gambiae*		KJ728740.1
	wAnga_Mali	*Anopheles gambiae*		MF944114.1
	wAnga_TZ	*Anopheles arabiensis*		MH596693, MH596696 MH596697, MH596703
Outgroup control	*Rickettsia*	*Rickettsia montanensis* ATCCVR-611	–	NR025920

### Data analysis

Statistical analyses and graph generation were performed using GraphPad Prism software version 5.0 (GraphPad Software, Inc., San Diego, CA, USA). Statistically significant differences were identified using the non-parametric Mann-Whitney test. A *p *value < 0.05 was regarded as being statistically significant.

## Results

### Amplification of the *Wolbachia* 16S rRNA-encoding gene from field-captured *Anopheles* species

In the initial PCR, the W-SpecF and W-SpecR primers specifically bound to a conserved region at the 3ʹ end of the *Wolbachia* 16S rRNA-encoding gene, generating an amplicon with an approximate length of 438 bp (Fig. 2a, upper panel). A representative image of agarose gel electrophoresis shows low-intensity DNA bands of between 400 and 500 bp, amplified from DNA pools of *An. minimus*, *An. maculatus*, and *An. peditaeniatus* (Fig. 2b). Among 73 DNA sample pools, 14 pools yielded 438-bp amplicons: 10 pools of *An. minimus*, 3 pools of *An. peditaeniatus*, and 1 pool of *An. maculatus*. Preliminary data of two pools of *An. aconitus* containing seven DNA samples in each pool yielded 538-bp amplicons in one pool (additional file 1). The W-Spec-based PCR product obtained from the 15 DNA sample pools from the initial runs was subsequently used as template in the nested PCR using the 16SN primer (Fig. 1a, lower panel). The 16SNF and 16SNR primers yielded amplicons from *An. minimus* pool numbers 21, 32, and 36 (Fig. 2c and Additional file 1: Fig. S1). Given that the size and intensity of the amplicon obtained from *An. minimus* pool numbers 36 and 40 were similar to that of the no template control or smaller than that of the positive control, respectively (Fig. 2c), we therefore interpreted this as a negative result. No amplification was observed in *An. peditaeniatus, An. maculatus*, or *An. aconitus* in the nested PCR (Additional file 1).

To confirm whether the yielded amplicons were the conserved region of *Wolbachia* 16S rRNA, the PCR products derived from DNA pool numbers 21 (P21) and 32 (P32) from *An. minimus* were subjected to DNA sequencing using 16SNF and 16SNR primers. The GenBank database was searched for similar sequences using the BLASTN program. DNA sequencing of P21 and P32 yielded 326 nucleotides, and all 326 nucleotides of P21 and P32 were aligned with 97 and 100 sequences, respectively, of *Wolbachia pipientis* 16S rRNA (query coverage = 100). P21 and P32 were 100% and 99.39% identical to all 16S rRNA sequences of *W. pipientis*, respectively. The BLASTN and MSAViewer results are provided as an Additional file 2. Hereafter, we referred to the *Wolbachia* strain identified in *An. minimus* as *w*Anmi. The place and pool number were tagged; *w*Anmi_UmpP21 and *w*Anmi_UmpP32 represent *Wolbachia* from *An. minimus* isolated from Umphang Valley and from pool numbers 21 and 32, respectively.

### Correlation of *Wolbachia* and *Plasmodium* infection in *Anopheles minimus*

**Fig. 3.**
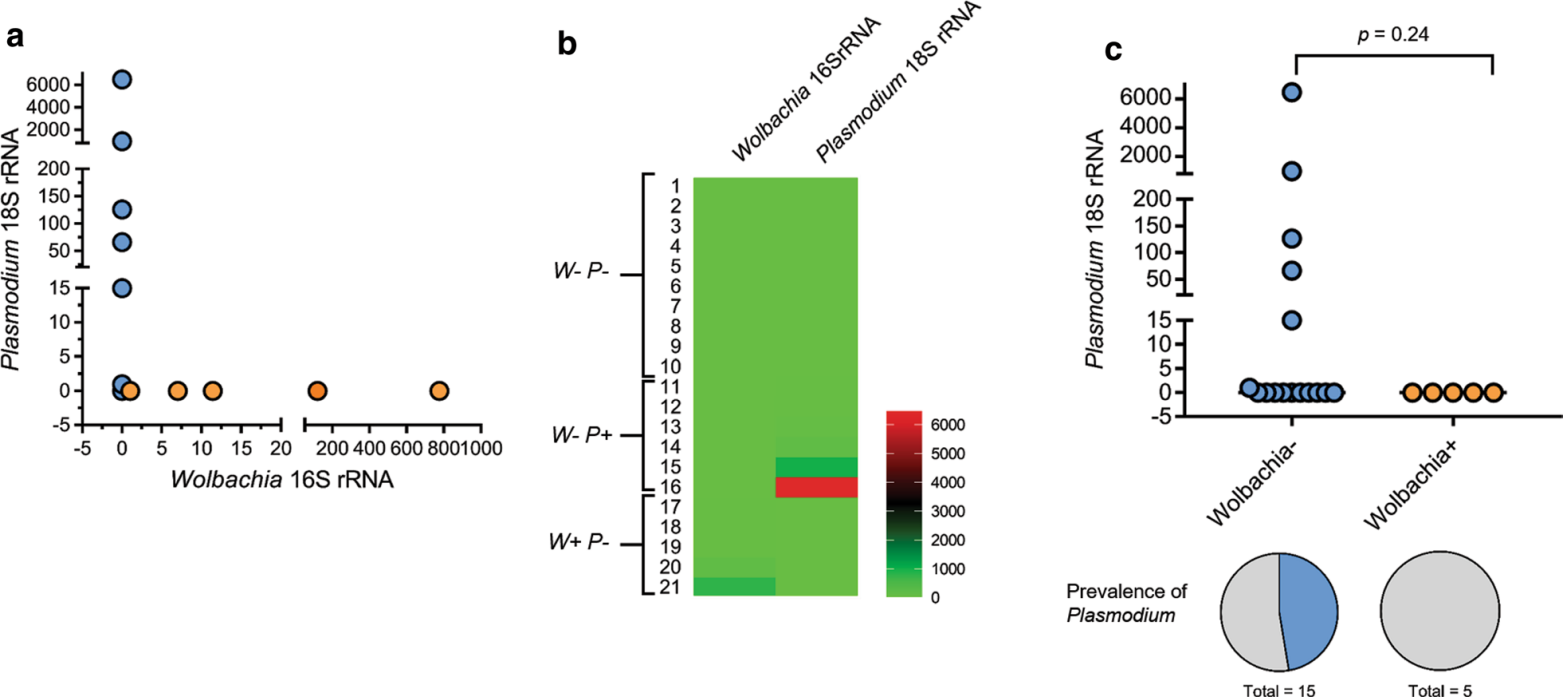
Correlation of *Wolbachia* and *Plasmodium* infection in *An. minimus*. Levels of *Wolbachia* 16S rRNA and *Plasmodium* 18S rRNA were examined using quantitative PCR. DNA levels in individual samples were normalized with that of *An. minimus* ITS2A. Levels of each DNA were relative to a reference sample, which was set as 1. **a** Relative levels of *Wolbachia* (*X* axis) and *Plasmodium* (*Y* axis) in the individual samples of *An. minimus* pool numbers 21 and 32 were plotted. Five DNA samples were detectable for *Wolbachia* 16S rRNA (orange dots), while the others were undetectable (blue dots). **b** Heatmap compares relative levels of *Wolbachia* 16S rRNA and *Plasmodium* 18S rRNA in all 21 *An. minimus* samples. High to low DNA levels were displayed as red to green, respectively. Numbers are relative levels calculated using the 2^−∆∆CT^ method. *W*: *Wolbachia* 16S rRNA-coding region; *P*: plasmodium 18S rRNA-coding region. **c** Level and prevalence of *Plasmodium* in the *An. minimus* DNA samples with and without *Wolbachia* (blue and orange dots, respectively)

Levels of *Wolbachia* and *Plasmodium* in the DNA sample of *An. minimus* were calculated based on the level of the respective 16S and 18S rRNA amplification using quantitative real-time PCR. Among 21 individual samples of pool number 21 (*n* = 10) and 32 (*n* = 11), five samples exhibited amplification of the *Wolbachia* 16S rRNA region (orange dots in Fig. 3a). The amplicons were detected at the cycle threshold of 32–39 and had the same melting temperature. After normalization with levels of the *An. minimus* ITS2A region, relative levels of *Wolbachia* 16S rRNA varied among the five samples: 7–776 fold differences (Fig. 3a). To examine correlation between *Wolbachia* and *Plasmodium* in the individual *An. minimus*, we deployed the heatmap to indicate the relative level of both microorganisms in the individual *An. minimus*. All *Wolbachia*-positive samples (*n* = 5) exhibited undetectable levels of *Plasmodium* 18S rRNA (Fig. 3b). By contrast, *Plasmodium*-positive *An. minimus* (*n* = 6) had undetectable levels of *Wolbachia*, implying negative correlation between *Wolbachia* and *Plasmodium* (Fig. 3b). However, the level and prevalence of *Plasmodium* in *An. minimus* samples were not statistically significantly different between the two groups: the undetectable and detectable *Wolbachia* (*p* = 0.71, Fig. 3c).

### Phylogenetic analysis

**Fig. 4:**
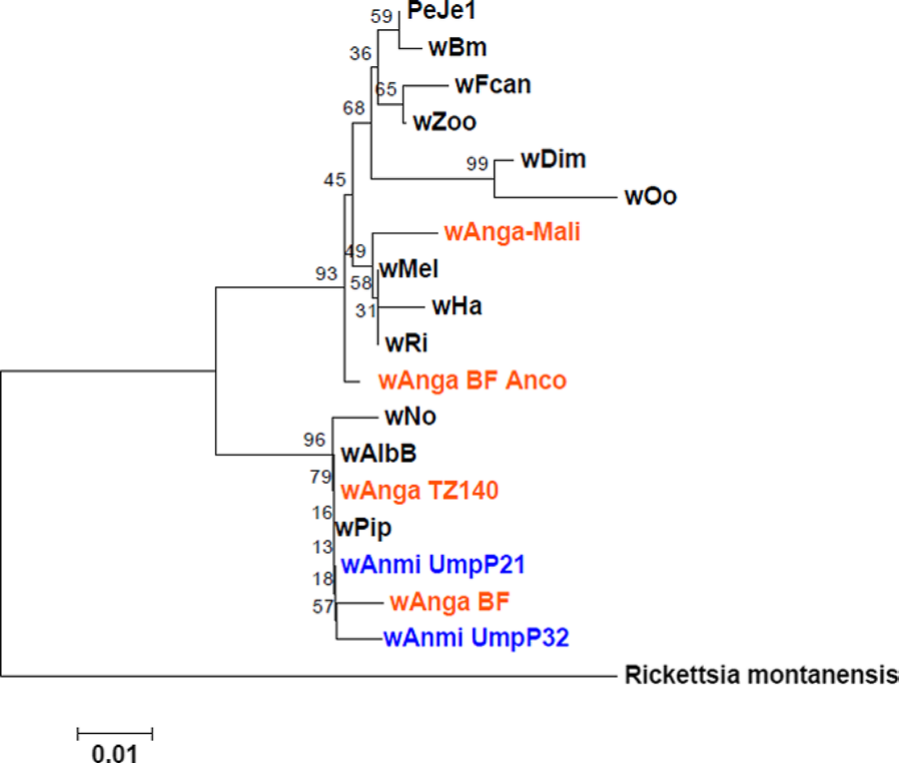
Rooted phylogenetic tree of the *Wolbachia* strain identified in *An. minimus* collected from Umphang Valley compared to other *Wolbachia* strains. The conserved region of the *Wolbachia* 16S rRNA-encoding DNA sequence obtained from the identified *Wolbachia* strains (blue letters) was phylogenetically compared with those in *Wolbachia* subgroups A, B, C, D, E, F, and H (black letters) and *Anopheles*-specific subgroups (orange letters) using MEGA software version 10. *Rickettsia montanensis* was used as the reference outgroup. A tree scale of 0.01 corresponds to inferred evolutionary changes. Details of the DNA sequences retrieved from GenBank are shown in Table 2

**Fig. 5:**
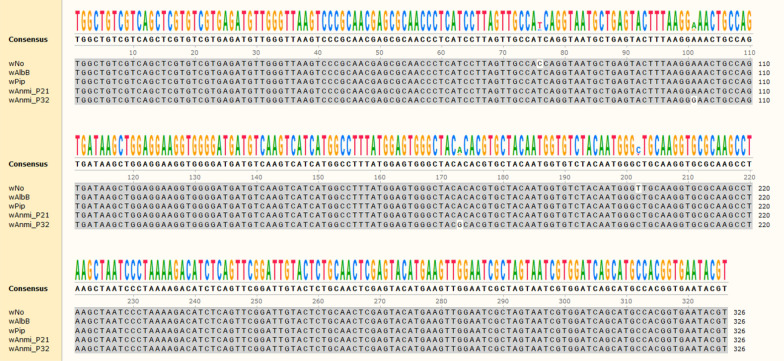
Multiple sequence alignment of the conserved region of the *Wolbachia* 16S rRNA gene. The sequence of the conserved region of the *Wolbachia* 16S rRNA gene was compared to those of wNo in *Drosophila simulans* (CP003883.1), *w*AlbB in *Aedes albopictus* (KX155506.1), and *w*Pip in *Culex quinquefasciatus* (AM999887.1), members of subgroup B. DNA sequences were aligned with the *w*Anmi_UmpP21 and *w*Anmi_UmpP32 strains identified in the present study. The consensus sequence was illustrated using MSAViewer

**Fig. 6:**
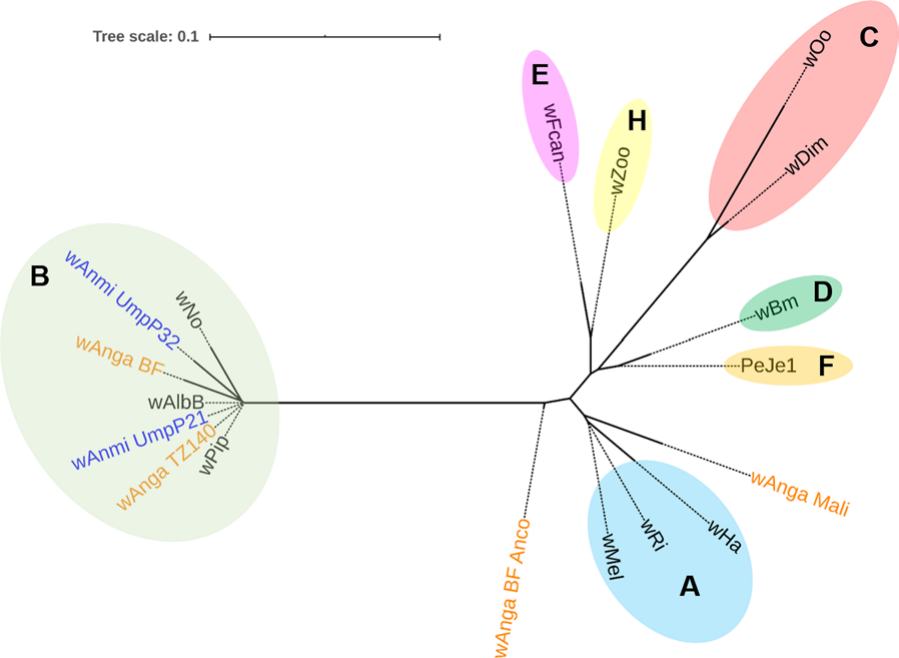
Unrooted phylogenetic tree of the *Wolbachia* strain identified in *An. minimus* collected from Umphang Valley compared to other *Wolbachia* strains. The conserved region of the *Wolbachia* 16S rRNA-encoding DNA sequence obtained from the identified *Wolbachia* strains (blue letters) was phylogenetically compared with those of *Wolbachia* subgroups A, B, C, D, E, F, and H (black letters) and *Anopheles*-specific subgroups (orange letters) using NGPhylogeny.fr. *Rickettsia montanensis* was used as the reference outgroup. A tree scale of 0.01 corresponds to inferred evolutionary changes. Details of the DNA sequences retrieved from GenBank are shown in Table 2

To assess the relationship of wAnmi_UmpP21 and wAnmi_UmpP32 with other known *Wolbachia* strains, we assembled a phylogenetic tree to determine genetic similarity and heterogeneity based on the conserved region of the *Wolbachia* 16S rRNA-encoding gene (Fig. 2a, c), a heritable region in prokaryotes. To validate the output data, we employed rooted and unrooted phylogenetic inference methods. Based on an assumption of a common ancestral path, the rooted maximum likelihood phylogram illustrated that both *w*Anmi_UmpP21 and *w*Anmi_UmpP32 were genetically related to *Wolbachia* subgroup B (Fig. 4). *w*Anm_UmpP21 was in the same cluster as the *w*Pip strain from *Cx. quinquefasciatus* and the *w*AlbB strain from *Ae. albopictus*. In contrast, *w*Anmi_UmpP32 was closely related to *w*Anga isolated from *An. gambiae* in Burkina Faso (wAnga_BF) and *w*Anga isolated from *An. arabiensis* in Tanzania (wAnga_TZ140) (Fig. 4). To reveal the extent of genetic similarity between the *Wolbachia* members in subgroup B, we aligned the *Wolbachia* 16S rRNA-conserved regions of the *w*No, *w*AlbB, *w*Pip, *w*Anmi_UmpP21, and *w*Anmi_UmpP32 strains (Fig. 5). One hundred percent similarity was observed among the *w*AlbB, *w*Pip, and *w*Anmi_UmpP21 strains. However, *w*Anmi_UmpP32 had two single nucleotide polymorphisms at two locations, implying genetic variation in *Wolbachia* in wild *An. minimus* in Umphang Valley. In agreement with the rooted maximum likelihood phylogenetic tree, excluding the assumption of a common ancestor, the unrooted, bifurcating phylogenetic tree revealed that *w*Anmi_UmpP21 and *w*Anmi_UmpP32 clustered in the leaf node of *Wolbachia* subgroup B, confirming a close genetic relationship (Fig. 6).

## Discussion

The data presented herein are preliminary evidence of native *Wolbachia* in *An. minimus*, a major malaria vector in an endemic area of mainland Southeast Asia. The identified *Wolbachia* in *An. minimus*, herein called wAnmi, was phylogenetically clustered in subgroup B, similar to *w*AlbB, which has been associated with the suppression of development of *P. falciparum* in *An. stephensi* [22]. Along the Thailand-Myanmar border, malaria transmission continues in many areas [46, 47], including the Umphang District of Tak Province in western Thailand. Most collection sites were in the Ban Nong Luang Village of Umphang District located in a valley primarily surrounded by forested mountains. Since some villagers engage in hunting-gathering and agriculture in areas near the forest (Fig. 1a), the risk of malaria infection in individuals is high. In the Umphang Valley, there are reportedly seven putative malaria vectors: *An. minimus*, *An. dirus*, *An. baimaii*, *An. sawadwongporni*, *An. maculatus*, *An. pseudowillmori*, and *An. aconitus* [30]. In the Thasongyang District northern Umphang Valley, female *An. minimus*, *An. maculatus*, *An. annularis*, and *An. barbirostris* have been shown to carry *P. vivax* sporozoites, confirming their role in malaria transmission [31]. Regarding the number of *Anopheles* in the Umphang Valley, *An. minimus* was the most abundant (> 50%), followed by the potential malaria vectors *An. peditaeniatus* (~ 20%) and *An. maculatus* (~ 10%) [30]. Hence, this study included samples from *An. minimus*, *An. peditaeniatus*, and *An. maculatus*, the major malaria vectors, for the detection of *Wolbachia*. In addition, we examined 14 DNA samples of An. aconitus samples as a preliminary data.

In Thailand, only one survey of *Wolbachia* in mosquitoes was conducted to amplify the *filamenting temperature-sensitive mutant Z* (*ftsz*) and *Wolbachia surface protein* (*wsp*) genes. All 23 mosquito species in the genera *Aedes*, *Culex*, and *Mansonia* were positive for the *ftsz* and *wsp* genes, whereas none of the 19 *Anopheles* species were positive [18]. Failure to detect *Wolbachia*-specific genes in *Anopheles* spp. was consistent with the results of studies in European, African, and American specimens [19, 20]. Nevertheless, detection of the *Wolbachia* 16S rRNA region was accomplished. The W-Spec primers were designed to specifically amplify a 438-bp sequence at the 3ʹ region of the 16S rRNA gene in *Wolbachia* [27]. The W-Spec primers allowed the detection of *Wolbachia* in temperate North American arthropods, including the family *Culicidae* but excluding other mosquito families. Subsequently, Baldini et al. reported the first evidence of *Wolbachia* in the reproductive organs of male and female *An. gambiae*, a major malaria vector in sub-Saharan Africa. In the same DNA samples, the W-Spec primer-based PCR was able to amplify the 16S rRNA fragment, whereas *Wolbachia*-specific surface protein and fructose-biphosphate aldolase-based PCR failed [23], implying good sensitivity of the W-Spec primers. Moreover, Shaw et al. further improved the sensitivity of W-Spec primer-based PCR by using nested primers (16SNF and 16SNR). The use of nested PCR allowed the detection of *Wolbachia* in *An. coluzzii* [25], *An. gambiae* in Mali [26], and *An. arabiensis* in Tanzania [28]. Additional studies were able to amplify the *Wolbachia* 16S rRNA fragment in DNA samples extracted from head-thorax or thorax-abdomen, implying the possibility of *Wolbachia* infection in nonreproductive organs [22, 29]. Collectively, *Wolbachia* infection in somatic and germ cells can be detected using nested PCR, which amplifies the conserved region of the *Wolbachia* 16S rRNA gene.

Nested PCR is regarded as a highly sensitive tool for detecting targets of interest that are present in very low amounts. We sometimes failed to amplify the 438-bp fragment using W-SpecF and W-SpecR in the initial PCR; however, there were 412-bp amplicons observed in the nested PCR [25], implying good sensitivity of the nested PCR. As such, false-negative results may occur in cases of low-intensity *Wolbachia* infection because the quantity of the target of interest is below the limit of nested PCR. Given that DNA samples were extracted from the head and thorax of female *Anopheles*, detection failure in nested PCR is possibly because of low-intensity infection or the reproductive organ specificity of *Wolbachia*. Therefore, assays with high sensitivity, such as quantitative PCR, may aid in the detection of low-intensity *Wolbachia* infection [39, 48, 49]. Moreover, DNA preparation from the whole body of mosquitoes ensures the inclusion of *Wolbachia* strains that specifically infect germ cells.

The high sensitivity of nested PCR may cause low specificity, especially when primers bind to the conserved region of a common gene. Since *Wolbachia* is capable of infecting the majority of insect species [50], and the W-Spec primers amplify the conserved region of the *Wolbachia* 16S rRNA-coding gene, false-positive results due to environmental contamination from other insects may occur. In our study, 438-bp DNA amplicons with low-fluorescence intensity were present in the initial PCR, but we failed to reamplify these amplicons in the subsequent nested PCR using the 16SNF and 16SNR primers, suggesting the possibility of nonspecific amplification in the initial run. Moreover, if the environmental contamination is at an extremely low level in the initial run and cannot be detected, the subsequent runs will be able to amplify, owing to a sufficient amount of template. As shown in Fig. 2c, the DNA band could be observed in the no template control. To minimize environmental contamination, PCR preparations were performed in a clean hood for the initial and nested PCR. Despite great care in the pre-PCR steps, we sometimes observed DNA bands in the negative control lane. Thus, DNA sequencing of the PCR product was necessary to confirm *Wolbachia*-specific amplification. Importantly, given the possible environmental contamination in the previous survey of *Anopheles* spp., a new field study of *Wolbachia* in *A. minimus* needs to be conducted in the same area. To address environmental contamination, the DNA probe- or antibody-based microscopic imaging of *Wolbachia* will be employed to validate the PCR-based findings. We are now undertaking the field study in the Umphang Valley. Because the additional work may take some time, we will report the finding in a new study.

To the best of our knowledge, the present data are the first preliminary evidence of native *Wolbachia* in *An. minimus*. However, this study has limitations. First, the presence of the *Wolbachia* 16S rRNA gene in DNA samples from *Anopheles* is not direct evidence of natural *Wolbachia* infection because environmental contamination during mosquito capture, DNA extraction, and PCR preparation is possible. Intracellular localization of *Wolbachia* in *Anopheles* spp. is a more definitive indicator of *Wolbachia* infection than sequencing. This could be done by using *in situ* hybridization [25, 51]. Moreover, given the availability of the remaining DNA samples obtained during a previous 2-year survey [30], this study included the DNA samples regardless of the sample size. Thus, the prevalence of *Wolbachia* in *Anopheles* spp. in Umphang Valley could not be statistically estimated. Finally, all mosquitoes were collected from the same area located in the Ban Nong Luang village, and only *Wolbachia* subgroup B was identified in *An. minimus*. By contrast, Sawasdichai et al. could molecularly detect the high diversity of *Wolbachia* in *An. minimus* and *An. maculatus* collected from different villages [39]. Thus, the *Wolbachia* subgroup B in *An. minimus* may represent a subpopulation of *Wolbachia*, and undetectable amplification of *Wolbachia* in *An. maculatus*, *An. peditaenitus*, and *An. aconitus* did not indicate that these species are refractory to *Wolbachia* infection. Detection of *Wolbachia* in more diverse areas will address this issue. Collectively, identification of the *Wolbachia* strain in *Anopheles* spp. requires further confirmation, in which high-sensitivity assays, such as fluorescent *in situ* hybridization [25] and quantitative PCR [39], whole mosquitoes, and more diverse areas will be included.

*Wolbachia* has been under investigation for its potential application in blocking malaria transmission. In a recent report, *An. gambiae* mosquitoes were naturally infected with *Wolbachia* at different levels, and those infected with a high level of *Wolbachia* were likely devoid of *Plasmodium* development [26]. In agreement with the study in *An. gambiae*, the relative level of *Wolbachia* varied among *An. minimus* examined in our study. The likelihood of *Plasmodium* inhibition was observed in the *Wolbachia*-deteced *An. minimus*; however, low sample numbers of *An. minimus* having *Wolbachia* 16S rRNA resulted in a non-significant difference in the prevalence and level of *Plasmodium*. Thus, more field-isolated *An. minimus* need to be included to provide a definitive tendency. As proof of concept, field trials in Australia demonstrated that the release of laboratory-reared mosquitoes infected with *Wolbachia* resulted in the rapid spread of *Wolbachia* among wild uninfected mosquito populations [15]. Population invasion by a particular *Wolbachia* strain depends on the level of cytoplasmic incompatibility, host fitness (survival, fecundity and fertility), and vertical transmission. Therefore, the following issues need to be assessed: the potential of the native *Wolbachia* identified in *An. minimus* to render resistance to *Plasmodium* parasites and interfere with malaria transmission, its ability to cause cytoplasmic incompatibility, and its effects on host fitness.

## Conclusion

To the best of our knowledge, the data presented herein are the first molecular evidence of a *Wolbachia* strain in *An. minimus*, named *w*Anmi, in a low-malaria transmission area in the Umphang Valley of western Thailand. Further biological characterization is required to examine its potential as a malaria transmission control strategy in the field.

## Supplementary information

**Additional file 1:** Results of the standard PCR.

**Additional file 2:** BLASTN result of P21. BLASTN result of P32.

## Data Availability

All data generated or analyzed during this study are included in this published article and its Additional information files. The sequences of *Wolbachia* 16S rRNA fragments generated in the present study were submitted to GenBank under the accession numbers MT449018 and MT449019 and are retrievable.

## Abbreviations

*An*.: Genus *Anopheles*
16S rRNA: Small subunit 16 of ribosomal ribonucleic acid
16SNF: Small subunit 16 of ribosomal ribonucleic acid-nested forward primer
16SNR: Small subunit 16 of ribosomal ribonucleic acid-nested reverse primer
Ump: Umphang
W-SpecF: Wolbachia-specific forward primer
W-SpecR: Wolbachia-specific reverse primer
