# Supplementary material for: Molecular identification of native Wolbachia pipientis in Anopheles minimus in a low-malaria transmission area of Umphang Valley along the Thailand-Myanmar border
Source: Parasit Vectors. 2020 Nov 16;13:579. doi: 10.1186/s13071-020-04459-7 (PMC7670599; doi:10.1186/s13071-020-04459-7)
Supplement: Supplementary file 1 — Additional file 1: Results of the standard PCR. [file 13071_2020_4459_MOESM1_ESM.doc]

**Additional information file**

**1. Results of the standard PCR**

For all positive PCR results, the 438-bp amplicon was present, and PCR amplification without template (no template control or NTC) did not yield a 438-bp amplicon (Figure 1-8). DNA pools from *An.* *minimus* (n = 40), *An. peditaeniatus* (n = 20)*, An. maculatus* (n = 13) and *An. aconitus* (n = 2) was subjected to PCR using the W-SpecF and W-SpecR primers. Ten DNA pools from *An.* *minimus*, 3 DNA pools from *An. peditaeniatus,* and 1 DNA pool each from *An. maculatus* and *An. aconitus* yielded 438-bp amplicons. The data are summarized in Table 1 of this Additional Information.


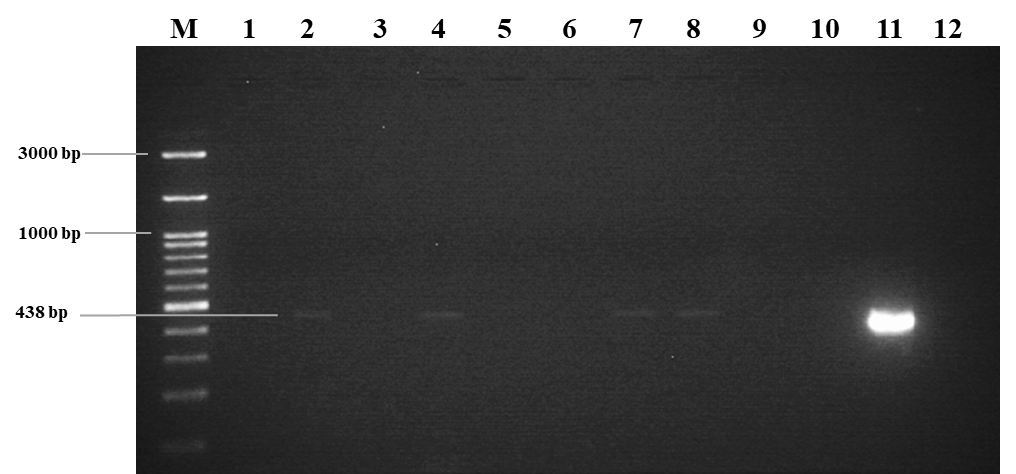


**Figure 1** Amplicons from the standard PCR were electrophoresed on a 2% agarose gel, stained with ViSafe Red and exposed to UV light. Each lane shows the PCR product from the respective DNA pool.

Lane M: DNA marker (100-bp ladder)

Lane 1: *An.* *minimus* pool no. 1

Lane 2: *An.* *minimus* pool no. 2

Lane 3: *An.* *aconitus* pool no. 1

Lane 4: *An.* *aconitus* pool no. 2

Lane 5: *An.* *maculatus* pool no. 1

Lane 6: *An.* *maculatus* pool no. 2

Lane 7: *An.* *maculatus* pool no. 3

Lane 8: *An.* *peditaeniatus* pool no. 1

Lane 9: *An.* *peditaeniatus* pool no. 2

Lane 10: *An.* *peditaeniatus* pool no. 3

Lane 11: *Mansonia* spp. (positive control)

Lane 12: Blank control


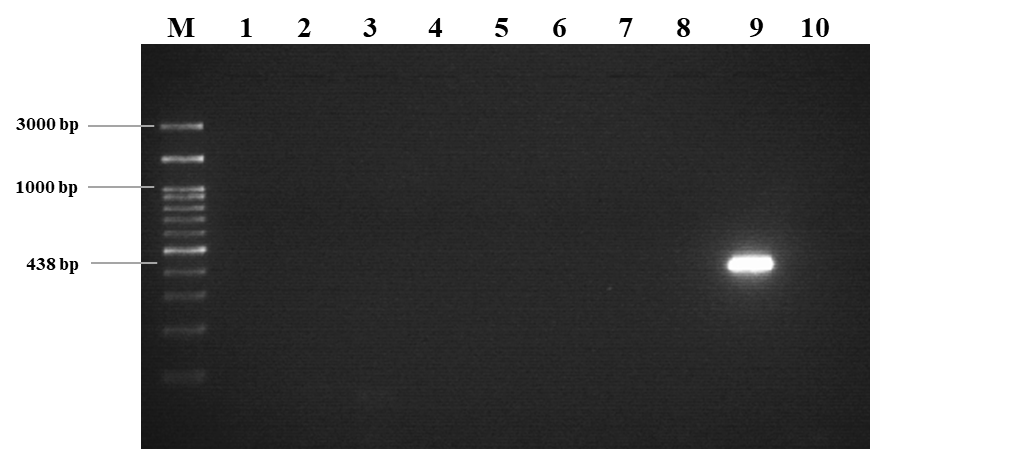


**Figure 2** Amplicons from the standard PCR were electrophoresed on a 2% agarose gel, stained with ViSafe Red and exposed to UV light. Each lane shows the PCR product from the respective DNA pool.

Lane M: DNA marker (100-bp ladder)

Lane 1: *An.* *minimus* pool no. 3

Lane 2: *An.* *minimus* pool no. 4

Lane 3: *An.* *minimus* pool no. 5

Lane 4: *An.* *minimus* pool no. 6

Lane 5: *An.* *minimus* pool no. 7

Lane 6: *An.* *minimus* pool no. 8

Lane 7: *An.* *maculatus* pool no. 4

Lane 8: *An.* *maculatus* pool no. 5

Lane 9: *Mansonia* spp. (positive control)

Lane 10: Blank control


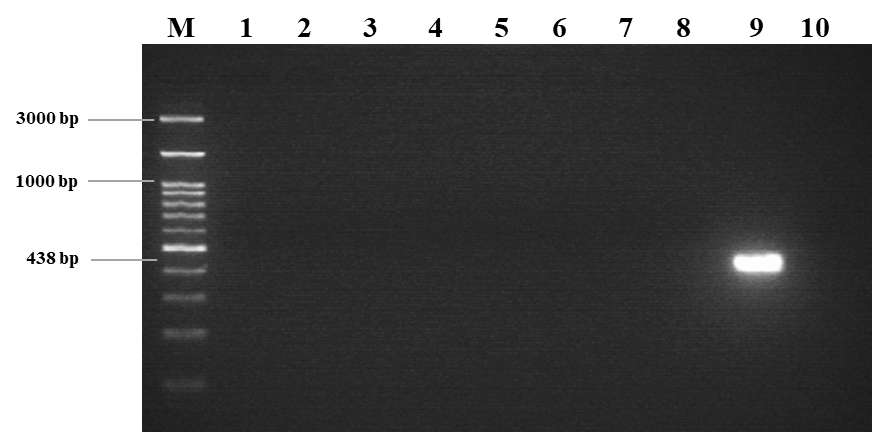


**Figure 3**  Amplicons from the standard PCR were electrophoresed on a 2% agarose gel, stained with ViSafe Red and exposed to UV light. Each lane shows the PCR product from the respective DNA pool.

Lane M: DNA marker (100-bp ladder)

Lane 1: *An.* *maculatus* pool no. 6

Lane 2: *An.* *maculatus* pool no. 7

Lane 3: *An.* *maculatus* pool no. 8

Lane 4: *An.* *peditaeniatus* pool no. 4

Lane 5: *An.* *peditaeniatus* pool no. 5

Lane 6: *An.* *peditaeniatus* pool no. 6

Lane 7: *An.* *peditaeniatus* pool no. 7

Lane 8: *An.* *peditaeniatus* pool no. 8

Lane 9: *Mansonia* spp. (positive control)

Lane 10: Blank control


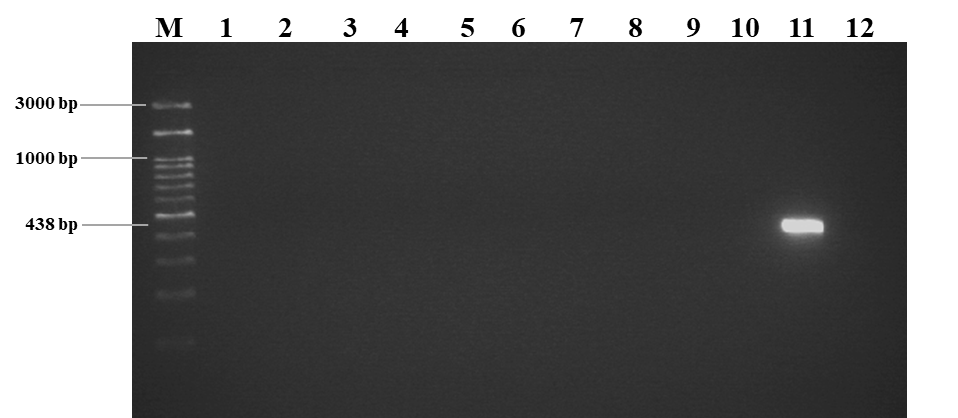


**Figure 4**  Amplicons from the standard PCR were electrophoresed on a 2% agarose gel, stained with ViSafe Red and exposed to UV light. Each lane shows the PCR product from the respective DNA pool.

Lane M: DNA marker (100-bp ladder)

Lane 1: *An.* *maculatus* pool no. 9

Lane 2: *An.* *maculatus* pool no. 10

Lane 3: *An.* *maculatus* pool no. 11

Lane 4: *An.* *maculatus* pool no. 12

Lane 5: *An.* *maculatus* pool no. 13

Lane 6: *An.* *peditaeniatus* pool no. 9

Lane 7: *An.* *peditaeniatus* pool no. 10

Lane 8: *An.* *peditaeniatus* pool no. 11

Lane 9: *An.* *peditaeniatus* pool no. 12

Lane 10: *An.* *peditaeniatus* pool no. 13

Lane 11: *Mansonia* spp. (positive control)

Lane 12: Blank control


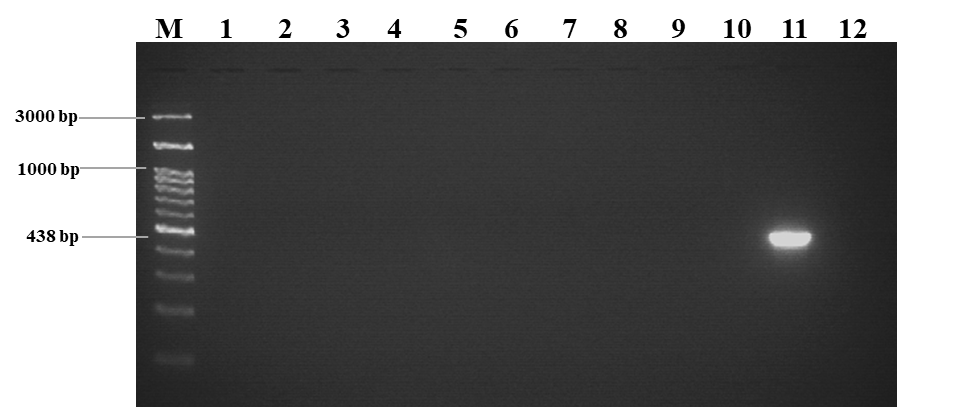


**Figure 5**  Amplicons from the standard PCR were electrophoresed on a 2% agarose gel, stained with ViSafe Red and exposed to UV light. Each lane shows the PCR product from the respective DNA pool.

Lane M: DNA marker (100-bp ladder)

Lane 1: *An.* *peditaeniatus* pool no. 14

Lane 2: *An.* *peditaeniatus* pool no. 15

Lane 3: *An.* *peditaeniatus* pool no. 16

Lane 4: *An.* *minimus* pool no. 9

Lane 5: *An.* *minimus* pool no. 10

Lane 6: *An.* *minimus* pool no. 11

Lane 7: *An.* *minimus* pool no. 12

Lane 8: *An.* *minimus* pool no. 13

Lane 9: *An.* *minimus* pool no. 14

Lane 10: *An.* *minimus* pool no. 15

Lane 11: *Mansonia* spp. (positive control)

Lane 12: Blank control


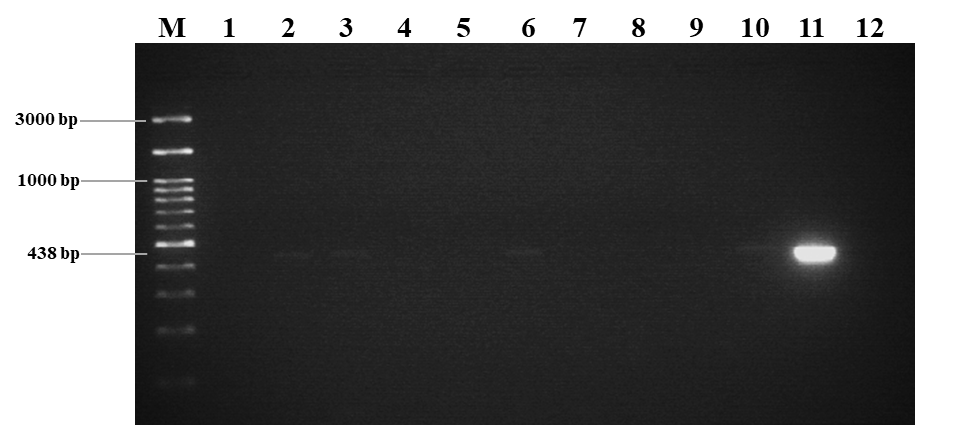


**Figure 6** Amplicons from the standard PCR were electrophoresed on a 2% agarose gel, stained with ViSafe Red and exposed to UV light. Each lane shows the PCR product from the respective DNA pool.

Lane M: DNA marker (100-bp ladder)

Lane 1: *An.* *peditaeniatus* pool no. 17

Lane 2: *An.* *peditaeniatus* pool no. 18

Lane 3: *An.* *peditaeniatus* pool no. 19

Lane 4: *An.* *peditaeniatus* pool no. 20

Lane 5: *An.* *minimus* pool no. 16

Lane 6: *An.* *minimus* pool no. 17

Lane 7: *An.* *minimus* pool no. 18

Lane 8: *An.* *minimus* pool no. 19

Lane 9: *An.* *minimus* pool no. 20

Lane 10: *An.* *minimus* pool no. 21

Lane 11: *Mansonia* spp. (positive control)

Lane 12: Blank control


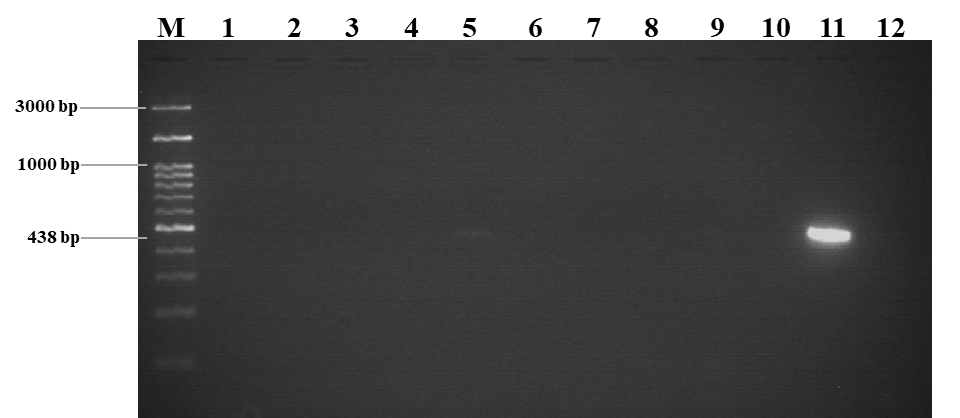


**Figure 7** Amplicons from the standard PCR were electrophoresed on a 2% agarose gel, stained with ViSafe Red and exposed to UV light. Each lane shows the PCR product from the respective DNA pool.

Lane M: DNA marker (100-bp ladder)

Lane 1: *An.* *minimus* pool no. 22

Lane 2: *An.* *minimus* pool no. 23

Lane 3: *An.* *minimus* pool no. 24

Lane 4: *An.* *minimus* pool no. 25

Lane 5: *An.* *minimus* pool no. 26

Lane 6: *An.* *minimus* pool no. 27

Lane 7: *An.* *minimus* pool no. 28

Lane 8: *An.* *minimus* pool no. 29

Lane 9: *An.* *minimus* pool no. 30

Lane 10: *An.* *minimus* pool no. 31

Lane 11: *Mansonia* spp. (positive control)

Lane 12: Blank control


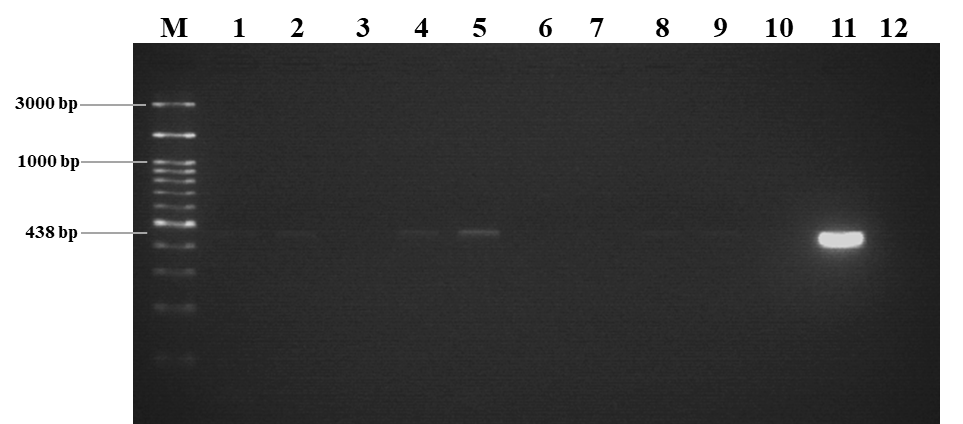


**Figure 8** Amplicons from the standard PCR were electrophoresed on a 2% agarose gel, stained with ViSafe Red and exposed to UV light. Each lane shows the PCR product from the respective DNA pool.

Lane M: DNA marker (100-bp ladder)

Lane 1: *An.* *minimus* pool no. 32

Lane 2: *An.* *minimus* pool no. 33

Lane 3: *An.* *minimus* pool no. 34

Lane 4: *An.* *minimus* pool no. 35

Lane 5: *An.* *minimus* pool no. 36

Lane 6: *An.* *minimus* pool no. 37

Lane 7: *An.* *minimus* pool no. 38

Lane 8: *An.* *minimus* pool no. 39

Lane 9: *An.* *minimus* pool no. 40

Lane 10: *An.* *vagus*

Lane 11: *Mansonia* spp. (positive control)

Lane 12: Blank control

**Table 1** Mosquito DNA samples tested for infection with *Wolbachia* using PCR

| **DNA Pool**  **No.** |  | | | |
| --- | --- | --- | --- | --- |
|  | ***An. minimus*** | ***An. peditaeniatus*** | ***An. maculatus*** | ***An. aconitus*** |
| 1 | - | + | - | - |
| 2 | + | - | - | + |
| 3 | - | - | + | ND |
| 4 | - | - | - | ND |
| 5 | - | - | - | ND |
| 6 | - | - | - | ND |
| 7 | - | - | - | ND |
| 8 | - | - | - | ND |
| 9 | - | - | - | ND |
| 10 | - | - | - | ND |
| 11 | - | - | - | ND |
| 12 | - | - | - | ND |
| 13 | - | - | - | ND |
| 14 | - | - | ND | ND |
| 15 | - | - | ND | ND |
| 16 | - | - | ND | ND |
| 17 | + | - | ND | ND |
| 18 | - | + | ND | ND |
| 19 | - | + | ND | ND |
| 20 | - | - | ND | ND |
| 21 | + | ND | ND | ND |
| 22 | - | ND | ND | ND |
| 23 | - | ND | ND | ND |
| 24 | - | ND | ND | ND |
| 25 | - | ND | ND | ND |
| 26 | + | ND | ND | ND |
| 27 | - | ND | ND | ND |
| 28 | - | ND | ND | ND |
| 29 | - | ND | ND | ND |
| 30 | - | ND | ND | ND |
| 31 | - | ND | ND | ND |
| 32 | + | ND | ND | ND |
| 33 | + | ND | ND | ND |
| 34 | + | ND | ND | ND |
| 35 | + | ND | ND | ND |
| 36 | + | ND | ND | ND |
| 37 | - | ND | ND | ND |
| 38 | - | ND | ND | ND |
| 39 | + | ND | ND | ND |
| 40 | + | ND | ND | ND |

ND = an experiment was not completed due to no DNA samples

**3. Results of the nested PCR**

For all positive PCR results, the 412-bp amplicon was present, and PCR amplification without template (no template control or NTC) did not yield a 412-bp amplicon (Figure 1-8) or yielded DNA bands with low fluorescence intensity (Figure 1-8). The 438-bp amplicons from the standard PCR were used as templates in the nested PCR. The nested PCR amplified 412-bp fragments. According to the results, *An.* *minimus* DNA pool numbers 21, 32 and 36 were positive (Figures 9-10).

**
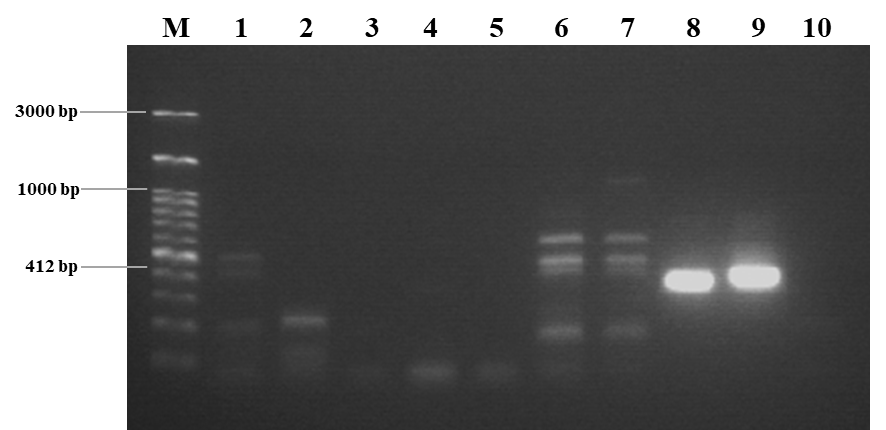
**

**Figure 9** Amplicons from the nested PCR were electrophoresed on a 2% agarose gel, stained with ViSafe Red and exposed to UV light. Each lane shows the PCR product from the respective DNA pool.

Lane M: DNA marker (100 bp ladder)

Lane 1: *An.* *aconitus* pool no. 2

Lane 2: *An.* *maculatus* pool no. 3

Lane 3: *An.* *peditaeniatus* pool no. 1

Lane 4: *An.* *peditaeniatus* pool no. 18

Lane 5: *An.* *peditaeniatus* pool no. 19

Lane 6: *An.* *minimus* pool no. 2

Lane 7: *An.* *minimus* pool no. 17

Lane 8: *An.* *minimus* pool no. 21

Lane 9: *Mansonia* spp. (positive control)

Lane 10: Blank control

**
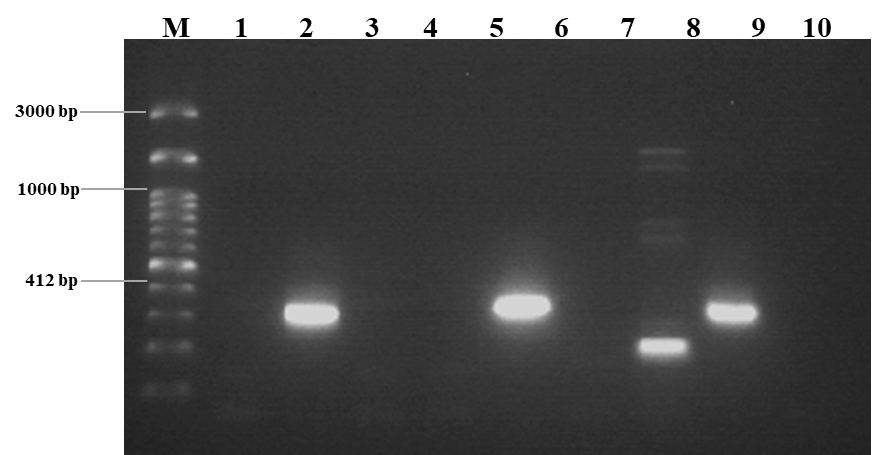
**

**Figure 10** Amplicons from the nested PCR were electrophoresed on a 2% agarose gel, stained with ViSafe Red and exposed to UV light. Each lane shows the PCR product from the respective DNA pool.

Lane M: DNA marker (DNA ladder)

Lane 1: *An.* *minimus* pool no. 26

Lane 2: *An.* *minimus* pool no. 32

Lane 3: *An. minimus* pool no. 33

Lane 4: *An.* *minimus* pool no. 35

Lane 5: *An.* *minimus* pool no. 36

Lane 6: *An.* *minimus* pool no. 39

Lane 7: *An.* *minimus* pool no. 40

Lane 8: *Mansonia* spp. (positive control)

Lane 9: Blank control
